# Observations on the Society for Widows, &c. &c.

**Published:** 1812-04

**Authors:** 


### *To the Editors of the Medical and Physical Journal.*

GENTLEMEN,

I BEG leave, through the medium of your Journal, to inform Mr. Leadbeater, p. 264, that it does not appear to have been the intention of the founders of "the Society for the Relief of the Widows and Orphans of Medical Men in London and its Vicinity," to admit among its members any others than such physicians, surgeons, and apothecaries, "as actually do reside and practise within the limits of the jurisdiction of the College of Physicians," i. e. within seven miles of London; and there seems to be a great degree of propriety in adopting this as a general rule; for, as no medical men but such as are regularly bred to the profession can be elected members, the society would not always be able to obtain from those at a distance a sufficient proof of their eligibility.

Such gentlemen, however, as have once been admitted, "may still continue members, and are entitled to all the privileges of the society," though they may remove beyond the limits of the society.

It may not, perhaps, be improper to repeat, what through your kindness I have been already allowed to say in your Journal, that the members of this society may be conveniently divided into three classes:

1st, Those who foresee a probability that their widows and orphans may require the future assistance of the society. Such men perform a very important duty towards their families by becoming members, for in no other society can they obtain the same advantages, on such easy terms.

2dly, Those whose families may possibly, but not very probably, be reduced to distress after the death of the father. These members are influenced, both by philanthropic and prudential motives, to belong to this society.

3dly, Those who, having risen so high in their profession as to bid defiance to indigence, are enabled from their plenty to afford assistance to their more unfortunate brethren. To the philanthropy of these more fortunate men, the society is chiefly indebted for the good it does. Of such men many are members of the society, and many who are not actually members, have distinguished themselves among its benefactors.

At however great a distance from London such *benefactors* may reside, their donations are most acceptable; and they may be assured that their liberality will not be misapplied.

I know not whether it becomes me here to add, that the directors of this society have to regret that their means of affording relief to the destitute widows and orphans, are still unequal to their wishes. I remain, Gentlemen,

Your humble Servant,

*Curzon-street, March* 10, 1812. S. M.

P. S. I take the liberty of referring Dr. Ramsbotham (p. 181) to Dr. S. H. Jackson's "Cautions to Women, &c." 1st edit. 1798, p. 157, where he will find the case of a child delivered by the forceps and brought to life "full half an hour" after the death of the mother. I do not remember to have seen any authentic case of a child's life having been preserved after a longer interval from the death of the mother, than Dr. Jackson has there recorded.

